# Complex Multimorbidity and Working beyond Retirement Age in Japan: A Prospective Propensity-Matched Analysis

**DOI:** 10.3390/ijerph19116553

**Published:** 2022-05-27

**Authors:** Daisuke Kato, Ichiro Kawachi, Naoki Kondo

**Affiliations:** 1 Department of Family Medicine, Mie University Graduate School of Medicine, Tsu 514-8507, Japan; 2 Department of Gerontological Evaluation, Center for Gerontology and Social Science, National Center for Geriatrics and Gerontology, Obu 474-8511, Japan; 3 Department of Social and Behavioral Sciences, Harvard T.H. Chan School of Public Health, Boston, MA 02215, USA; ikawachi@hsph.harvard.edu; 4 Department of Social Epidemiology, Graduate School of Medicine and Public Health, Kyoto University, Kyoto 606-8315, Japan; kondo.naoki.0s@kyoto-u.ac.jp

**Keywords:** multimorbidity, complex multimorbidity, employment

## Abstract

Background: With the aging of populations worldwide, the extension of people’s working lives has become a crucial policy issue. The aim of this study is to assess the impact of complex multimorbidity (CMM) as a predictor of working status among retirement-aged adults in Japan. Methods: Using a nationwide longitudinal cohort study of people aged over 65 who were free of documented disability at baseline, we matched individuals with respect to their propensity to develop CMM. The primary outcome of the study was working status after the six-year follow-up. Results: Among 5613 older adults (mean age: 74.2 years) included in the study, 726 had CMM and 2211 were still working at the end of the follow-up. In propensity-matched analyses, the employment rate was 6.4% higher in the CMM-free group at the end of the six-year follow-up compared to the CMM group (725 pairs; 29.5% vs. 35.9%; *p* = 0.012). Logistic regression analysis showed that CMM prevented older people from continuing to work beyond retirement age and was a more important factor than socioeconomic factors (income or educational attainment) or psychological factors (depressive symptoms or purpose in life). Conclusions: Our study found that CMM has an adverse impact on the employment rate of older adults in Japan. This finding suggests that providing appropriate support to CMM patients may extend their working lives.

## 1. Introduction

Due to medical and public health advances throughout the world, people are not only living longer but also suffering from the simultaneous occurrence of multiple diseases (multimorbidity: MM). The combination of longer lives and a higher prevalence of MM will pose a serious challenge to healthcare and to social security system solvency [1]. Combined with the declining fertility rate, these trends are projected to lead to higher dependency ratios and a social security budgetary crisis in the future [2]. This means that governments will be forced to make unpopular choices, such as raising taxes, reducing social security benefits, or increasing the retirement age.

Increasing the retirement age is dependent on having a fit workforce. Because mortality is declining faster than disease prevalence, the number of people living with comorbidities is increasing throughout the world [3]. People are currently expected to live approximately 20 years after retirement age, many of them with MM. This makes it critical to understand the relationship between MM and the ability of older people to extend their working lives.

Conventionally, MM has been defined by the co-occurrence of two or more diseases [4], which sometimes highlights its limitations as an index due to its higher prevalence ratio (55–98%) [5,6]. In this study, we adopted a potentially more sensitive concept of MM, namely, complex multimorbidity (CMM) [7], which is defined as the involvement of three or more body system disorders (as opposed to a simple count of comorbid diseases).

CMM has been established as a risk factor for death and long-term care needs [8,9,10,11]. Furthermore, while employment status is strongly influenced by socioeconomic status (SES), older adults with CMM in particular tend to have lower SES than those with MM [8,12]. These findings demonstrate the significance of focusing on body systems and CMM, especially in MM studies focusing on SES.

The purpose of this study is to clarify the impact of CMM on the working status of retirement-aged adults in Japan.

## 2. Materials and Methods

### 2.1. Study Participants

We used panel data from three survey waves (2010, 2013, and 2016) of the Japan Gerontological Evaluation Study (JAGES), a nationwide cohort of older adults in Japan [13]. The compulsory Japanese residential registry system was used as the sampling frame. Self-administered questionnaires were mailed to all eligible older people in the case of municipalities with fewer than 5000 residents or by random sampling in the case of municipalities with more than 5000 residents. JAGES focused on community-dwelling individuals aged 65 years or older who were free of disability at baseline. The baseline survey in 2010 included 95,827 older adults from 16 municipalities.

**Figure 1:**
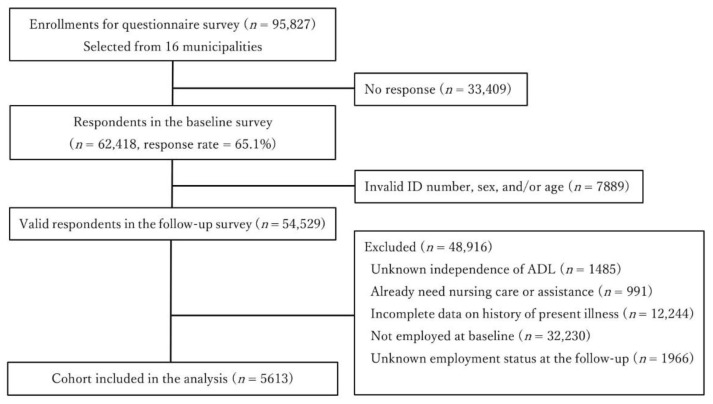
Participant flow diagram.

In total, 62,418 individuals answered the questionnaire (response rate: 65.1%). We excluded individuals who were not functionally independent, receiving any nursing care or home care assistance, or out of work at baseline. After excluding individuals with incomplete data on the history of present illnesses or missing information on employment status at follow-up, a cohort of 5613 individuals was identified for analysis (Figure 1). The most common reason for being excluded in our analysis was that the participant was already retired or not working at baseline (*n* = 32,230).

### 2.2. Measurements

#### 2.2.1. Complex Multimorbidity

At baseline, JAGES inquired about the presence of 19 diseases/health conditions. From this list, two symptoms, “difficulty swallowing” and “difficulty with bowel movement”, were excluded because they did not refer to specific diagnoses. Thus, the study included 17 diseases in total (Table 1), which were then classified into 9 categories according to the affected body system. CMM was defined as the coexistence of three or more body system disorders.

#### 2.2.2. Outcome

The outcome of the study was the employment status of older people after a six-year follow-up period. A questionnaire was administered, and participants replied whether they were in employment at that time. The type of employment (i.e., full-time, part-time, etc.) was not surveyed.

#### 2.2.3. Equivalized Income

Equivalized income was calculated by dividing household income (Japanese yen; JPY) by the square root of the number of people in the household and categorizing it into quartiles (first quartile: JPY 1.25 million per year and lower; second quartile: JPY 1.251–1.944 million per year; third quartile: JPY 1.945–3.061 million per year; and fourth quartile: JPY 3.062 million per year and higher). The first quartile is roughly equal to the Japanese relative poverty line during the JAGES survey period (JPY 1.22 million per year and lower) [14]. JPY was nearly equal to USD 0.01 during the JAGES survey period.

#### 2.2.4. Psychological Factors

To consider the psychological aspects of workers, two variables, ‘ikigai’ and the Geriatric Depression Scale (GDS), were introduced. “Ikigai” refers to the Japanese concept of purpose in life [15,16], which is a facet of positive psychology. It was asked about on the JAGES survey in 2013. Many Japanese workers find their purpose in life through work [17], and ‘ikigai’ has been linked with social participation and reduced mortality among older people [18,19].

The GDS is a psychometrically validated scale used to assess depressive symptoms; the scale consists of 0–15 points, which we then categorized into three groups: not depressed (0–4), mild depression (5–9), and severe depression (10–15) [20].

#### 2.2.5. Covariates

As potential confounders, we selected the following variables: age (in years); sex; marital status (married, widowed, separated, unmarried, or other); educational attainment (5 years or fewer, 6–9 years, 10–12 years, 13 years or more, or other); equivalized income (see above); smoking status (nonsmoker, current smoker, or ex-smoker); alcohol consumption (non-drinker, drinker, or ex-drinker); living alone (yes/no); engagement in leisure activities (yes/no); “ikigai”; depressive symptoms (not depressed, mild depression, or severe depression); and higher-level functional capacity measured by the Tokyo Metropolitan Institute of Gerontology Index of Competence (TMIG-IC), a 13-point scale [21].

### 2.3. Statistical Analysis

#### 2.3.1. Missing Data

Based on the missing-at-random assumption, we conducted multiple imputations for missing data. We analyzed 20 multiply imputed datasets using a bootstrapping expectation-maximization algorithm and combined all estimators using Rubin’s rule [22,23].

#### 2.3.2. Propensity Score Matching

We used propensity score matching to compare the employment rate among older adults with and without CMM. To address potential confounding bias, we conducted propensity score matching within the logistic regression model. The propensity score for each individual (i.e., the propensity to develop CMM) was estimated from the following variables: age, sex, marital status, educational attainment, equivalized income, smoking status, alcohol consumption, living alone, engagement in leisure activities, “ikigai”, depressive symptoms, higher-level functional capacity, and municipality code. We conducted a 1:1 matching between participants with and without CMM using the nearest-neighbor method using a caliper width of 0.2 of the standard deviation of the logit of the propensity score [24,25]. An absolute standardized difference greater than 0.1 was considered a significant imbalance of covariates between the groups (Table 2) [26].

Furthermore, we examined the impact of an alternative MM definition, that is, the conventional approach of counting the number of diseases [4]. While CMM signifies that three or more body systems are impaired by diseases, the conventional approach simply provides a tally of diseases. Using the conventional approach, we assessed the difference in the direction and significance of the results of analysis when participants had two or more diseases.

Lastly, we compared the effect size of CMM on working status with other predictors of employment, including SES (income and educational attainment), by using logistic regression analysis. Previous studies have shown that low SES is a risk for retiring before the statutory retirement age [27,28]. We adjusted for age (in years), sex, marital status (married or widowed/separated/unmarried/other), smoking status (nonsmoker or current smoker/ex-smoker), alcohol consumption (non-drinker or drinker/ex-drinker), living alone (yes/no), engagement in leisure activities (yes/no), “ikigai” (yes/no), depressive symptoms (not depressed, mild depression, and severe depression), and higher-level functional capacity by TMIG-IC [21].

All statistical analyses were conducted using the R software package (version 4.0.1). Statistical significance was set at *p* < 0.05.

## 3. Results

### 3.1. Baseline Population Characteristics

Among the study participants, 12% of men and 14% of women had CMM. With age, the prevalence of CMM increased, with 13% of the total population and 18% of those aged 75 years and older suffering from CMM (Table 3). A total of 18% of participants with CMM and 16% of those without CMM reported incomes at or below levels corresponding to the relative poverty threshold in Japan. Approximately half of the individuals with and without CMM had more than nine years of education.

### 3.2. Outcome

After 1:1 propensity score matching, 725 pairs were included in the analysis. The C-statistics before the matching for evaluation of the discriminatory ability of the propensity score model was 0.65 (95% confidence interval (CI): 0.63–0.67) [29]. The populations with CMM had a 6.4% lower employment rate than those without CMM after the six-year follow-up (29.5% vs. 35.9%; *p* = 0.012; odds ratio (OR), 1.33 (95% CI: 1.08–1.67)). In addition, the conventional MM definition (2+ diseases) did not influence the employment rate (37.2% vs. 39.6%; *p* = 0.114; OR, 1.10 (95% CI: 0.98–1.25)).

Table 4 shows the impact of risk factors for discontinuing work beyond the retirement age and contrasts the results from logistic regression analysis and propensity-score-matched analysis. The odds ratio for CMM was 1.35 (95%CI: 1.14–1.61). Meanwhile, the OR for income was 1.07 (95% CI: 1.02–1.13) and that for educational attainment was 1.02 (95% CI: 0.94–1.10). The absence of multicollinearity was confirmed, and the interaction term for each variable was not statistically significant.

## 4. Discussion

Although the employment of older people has been shown to contribute to improved health outcomes, such as lower mortality [30] and dementia [31], the present study sought to test the reverse hypothesis, namely, that MM in older people truncates their working lives. To the best of our knowledge, this is the first study to show that CMM reduces the employment rate of older people. Our finding implies that providing appropriate support to patients with CMM may extend their working lives.

In addition, we examined the effect of lower educational attainment and low income on the continued employment of older people and found that low income at baseline was a risk factor for continuing to work after retirement age, although it was not as influential as CMM (most (Q4) vs. least (Q1) income; OR: 1.20; 95% CI: 1.01–1.43). As for the educational background, there was no statistically significant association with working beyond retirement age, although this needs to be tested with a larger sample size. Economic disparities among older adults are likely to be amplified by disparities in employment status, given the incomes earned from work. That is, providing appropriate support to patients with CMM may be effective in not only extending their working life but also in reducing economic disparities among older people.

In contrast with previous reports from European countries, depressive symptoms among Japanese older people tended to be exacerbated by retirement [17], while employment among older people was associated with “ikigai” (higher sense of purpose in life). [15] However, even after controlling for both “ikigai” and depressive symptoms, CMM continued to be an independent predictor of working beyond the retirement age.

Further research is needed to examine factors that present barriers for older people from continuing to work. The impact of MM on working beyond retirement age will vary according to national differences in social policies (e.g., access to healthcare, legal protections for people with disabilities, and income security in retirement), as well as cultural variations in the level of social support available to individuals as they approach retirement age. For example, given the same level of MM, an individual living in a society with low-income security in retirement may be forced to continue working beyond retirement age compared to someone living in a society with more generous levels of social security.

In Japan, approximately 40% of older people hope to work throughout their lives, and approximately 80% of them hope to work until they are 70 or older [32]. In addition, the physical fitness test results of older people in their early 70s today are comparable to those of older people in their late 60s two decades ago [33]. As a result, the image of aging is changing throughout society. These data may suggest that extending the retirement age is a realistic possibility. Currently, the retirement age in Japan is 65, which means that the ratio of the working-age population (18–64) to the elderly (65+) is 2:1, meaning that 2:1 working-aged people support one older person. In contrast, if the retirement age is set at 75, the ratio will be 2:4 (if not extended, 1:3) in 2065 [34]. Therefore, if the retirement age can be extended by 10 years, the burden placed on the working-age population by older people can be reduced, even compared to the current situation.

Work is also beneficial to older people in other ways besides promoting good health. First, an occupation provides a social role and a social identity, such that the inability to continue working has a negative impact on people’s social lives that is distinct from the economic impact [35]. Second, increased income from continued employment may contribute to material health investments, which may further promote health among the elderly. Among the G7 countries, Japan has the highest employment rate among the elderly and the lowest “future replacement rate”, which refers to pension benefits divided by take-home pay before retirement (38.7%) [36]. While pension reform is necessary, it is also worthwhile to encourage older people to continue their work at the same time. Finally, ill-health due to disease may reduce the willingness of workers to acquire job skills. In other words, appropriate interventions in CMM could lead to an increase in a healthy and skilled workforce, ultimately leading to higher productivity and improved human capital, including health.

There are several limitations to the current study. First, in the current study, we did not consider the occupational class. However, we controlled for other aspects of socioeconomic status, including income and education [37]. Furthermore, in stratified analysis, we found that neither lower income nor education level modified the association between CMM and working status among older people.

Second, work desire was not considered among the reasons why older individuals chose to continue working beyond retirement age. In other words, the cohort analyzed in this study is a heterogeneous group, with a mix of people who continued to work because they wanted to and people who were forced to work for economic reasons. However, in this study, we found that higher income significantly contributed to the higher employment rate, which implies that the reason for older people to continue working was not due to financial needs.

Third, in this study, we adopted the CMM concept of impairment across body systems to define MM. For example, while the conventional approach to defining MM would consider “stroke” and “heart attack” to be separate diseases (yielding an MM score of 2), the CMM approach collapses them under the single entity, “disorders affecting the circulatory system.” Whilst we believe that the CMM approach better captures the common etiological pathways underlying co-occurring diseases (as well as their impact on clinical care), the method is strongly constrained by the data collected by the researchers. In JAGES, only 17 diseases were queried on the survey, and as shown in Table 1, among the nine body systems, four of them had only one disease representing that system. However, CMM inhibited older people from continuing to work beyond retirement age more than the conventional definition of MM (2+ diseases).

Finally, although our analysis was based on a longitudinal study design, this study is observational. Therefore, caution is needed when inferring causality.

The challenge of maintaining the fiscal solvency of social security systems has forced governments throughout the world to adopt measures such as raising taxes, reducing benefits, and raising the retirement age. Raising the age at which workers become eligible for benefits means that workers are often also family caregivers at the same time (e.g., taking care of sick parents or spouses) [38]. Therefore, for older people to continue to work, it is important to promote the diversification of work patterns, including part-time as well as full-time work. In future research, it is necessary to conduct an analysis that takes the type of work into account.

## 5. Conclusions

CMM had a negative impact on the employment rate of older adults in Japan. Extending the working lives of older people will require appropriate support for individuals with CMM.

## Figures and Tables

**Table 1:** Definition of CMM.

Body System Disorders	Surveyed Diseases
Circulation disorder	Heart disease (including arrhythmia)
	Stroke
	High blood pressure
Endocrine-metabolic disorder (General system)	Diabetes (including mild type)
	Obesity
	Dyslipidemia
Eye disorder	Impaired vision
Gastrointestinal disorder	Gastrointestinal disease
	Liver disease
Hearing disorder	Impaired hearing
Mental and behavioral disorder	Mental disease
	Sleep problem
Musculoskeletal and connective disorder	Osteoporosis
	Joint disease/neuralgia
	lnjury/fracture
Neoplasm	Cancer
Respiratory disorder	Respiratory disease

Abbreviation: CMM, Complex multimorbidity.

**Table 2:** Standardized mean differences before and after propensity score matching.

	SMD in Multiply Imputed Data	SMD In Matching Data
Characteristic		
Age	0.328	0.018
Sex	0.068	<0.001
Marriage	0.058	0.003
Single life	0.019	0.023
TMIG-IC	0.241	0.043
Alcohol consumption	0.077	0.061
Smoking status	0.005	0.047
Engagement for leisure activities	0.045	0.055
Educational attainment	0.072	0.019
Equivalized income	0.051	0.052
Ikigai	0.047	0.027
GDS score	0.358	0.069
Municipality code	0.005	0.013

Abbreviations: GDS, Geriatric Depression Scale; SMD, Standardized mean difference; TMIG-IC, Tokyo Metropolitan Institute of Gerontology Index of Competence.

**Table 3:** Characteristics of participants at baseline and employment rate after 6 years.

		With CMM (*n* = 726)	Without CMM (*n* = 4887)
Age—no. (%)	65–69	224 (31)	2086 (43)
	70–74	267 (37)	1616 (33)
	75–79	282 (39)	749 (15)
	80 and above	269 (37)	436 (9)
Sex—no. (%)	Male	419 (58)	2983 (61)
	Female	307 (42)	1904 (39)
Marital status—no. (%)	Married	554 (76)	3881 (79)
	Widowed	132 (18)	710 (15)
	Separated	15 (2)	173 (4)
	Unmarried	10 (1)	58 (1)
	Others	2 (0)	25 (1)
	Missing	13 (2)	40 (1)
Single life—no. (%)	Yes	647 (89)	4385 (90)
	No	71 (10)	446 (9)
	Missing	8 (1)	56 (1)
Mean TMIG-IC score (range)		11.5 (1–13)	11.8 (0–13)
	Missing—no. (%)	88 (12)	467 (10)
Alcohol consumption—no. (%)	Nondrinker	379 (52)	2356 (48)
	Drinker/Ex-drinker	304 (42)	2252 (46)
	Missing	43 (6)	279 (6)
Smoking status—no. (%)	Nonsmoker	350 (48)	2324 (48)
	Current smoker/Ex-smoker	313 (43)	2156 (44)
	Missing	63 (9)	407 (8)
Engagement for leisure activities—no. (%)	Yes	367 (51)	2644 (54)
	No	291 (40)	1900 (39)
	Missing	68 (9)	343 (7)
Educational attainment—no. (%)	5 or fewer	11 (2)	37 (1)
	6–9	328 (45)	2151 (44)
	10–12	249 (34)	1691 (35)
	13 or more	119 (16)	944 (19)
	Others	6 (1)	26 (1)
	Missing	24 (3)	75 (2)
Equivalized income ^a^—no. (%)	Q1: Very Low	128 (18)	758 (16)
	Q2: Low	132 (18)	937 (19)
	Q3: High	151 (21)	1026 (21)
	Q4: Very High	215 (30)	1618 (33)
	Missing	100 (14)	548 (11)
Ikigai—no. (%)	Yes	94(13)	676(14)
	No	15(2)	77(2)
	Missing	617(85)	4134(85)
Depressive symptoms (GDS score)—no. (%)	Non-depressed (0–4)	392(54)	3418(70)
	Mild depression (5–9)	175(24)	692(14)
	Severe depression (10–15)	52(7)	150(3)
	Missing	107(15)	627(13)
Employed at follow-up—no. (%)	Yes	214 (29)	1997 (41)
	No	512 (71)	2890 (59)

Abbreviations: CMM, Complex multimorbidity; GDS, Geriatric Depression Scale; TIMG-IC, the Tokyo Metropolitan Institute of Gerontology Index of Competence. ^a^ Income quartile calculated by all participants in the JAGES2010-2016 panel (‘Very Low’–1.250; ‘Low’ 1.251–1.944; ‘High’ 1.945–3.061; ‘Very High’ 3.062–million yen per year).

**Table 4:** Risk of discontinuation of working beyond retirement age.

	Unmatched Analysis with Multivariable Adjustment	Propensity Score Matched Analysis
	Odds Ratio for Discontinuation of Working beyond Retirement Age (95% CI)	
CMM	1.35 (1.14–1.61) *	1.33 (1.08–1.67) *
Educational attainment ^a^	1.02 (0.94–1.10)	
Equivalized income ^b^	1.07 (1.02–1.13) *	

*p*-values were calculated by using a generalized linear model. The reference group was (1) “5 or fewer years of education” in educational attainment and (1) “Low” in equivalized income. * *p* < 0.05. Abbreviations: CI, Confidence interval; CMM, Complex multimorbidity; GDS, Geriatric Depression Scale; ^a^ Educational attainment was classified into five categories: (1) 5 years or fewer, (2) 6–9 years, (3) 10–12 years, (4) 13 years or more, and (5) other. ^b^ Income quartile calculated by all participants in the JAGES2010-2016 panel (‘Very Low’ –1.250; ‘Low’ 1.251–1.944; ‘High’ 1.945–3.061; ‘Very High’ 3.062– million yen per year).

## Data Availability

The datasets of the Japan Gerontological Evaluation Study (JAGES), which were used in this research, are available from the corresponding author upon reasonable request. All inquiries should be addressed to the data management committee via e-mail: dataadmin.ml@jages.net. All JAGES datasets have ethical or legal restrictions for public deposition due to the inclusion of sensitive information from the human participants. Following the regulations of local governments which cooperated in the survey, the JAGES data management committee has imposed restrictions upon the data.
